# Characterization of Delirium in an Intensive Care Unit: Real-World Data Based on the Application of the Confusion Assessment Method

**DOI:** 10.7759/cureus.115982

**Published:** 2026-09-09

**Authors:** Francisco Teixeira da Silva, Ana Afonso, Joana M Esteves, Vitor Costa, Inês L Freitas, Ana Rita Branco, Rogério Corga da Silva

**Affiliations:** 1 Critical Care Medicine, Unidade Local de Saúde do Alto Minho, Viana do Castelo, PRT

**Keywords:** cam-icu, critical illness, delirium, intensive care unit, screening

## Abstract

Introduction

Delirium is a neuropsychiatric syndrome of acute onset characterized by disturbances in attention, awareness, and cognition. It is common in intensive care units (ICUs), and the Confusion Assessment Method for the ICU (CAM-ICU) is the most widely used bedside instrument for its detection.

Methods

We conducted a retrospective, observational, single-centre study of all patients admitted to the ICU of a Portuguese community hospital, Unidade Local de Saúde do Alto Minho (ULSAM), between January 1, 2021, and December 31, 2022. Delirium was defined as at least one positive CAM-ICU assessment during the ICU stay. Sociodemographic data, comorbidities, admission characteristics, severity scores (Acute Physiology and Chronic Health Evaluation II (APACHE II), Simplified Acute Physiology Score (SAPS), Sequential Organ Failure Assessment score (SOFA)), and outcomes were compared between patients with and without delirium using chi-square, Mann-Whitney U, and multivariable logistic regression.

Results

Of 788 ICU admissions (742 patients), 66 (8.4%) had at least one positive CAM-ICU assessment. Patients with delirium were older (median 71 vs 68 years, *p*=0.021) and had higher APACHE II, SAPS, and SOFA scores, longer ICU stay (median 8 vs 3 days, *p*<0.001), and a higher rate of invasive mechanical ventilation among those with known ventilation status (n=40/64, 62.5% vs n=279/692, 40.3%, *p*=0.001) than patients without delirium. In multivariable logistic regression, older age (OR 1.03, p=0.017) and male sex (OR 3.09 relative to female, p=0.001) were independently associated with delirium.

Conclusions

Delirium affected a modest but clinically important proportion of ICU admissions in this cohort and was associated with older age, male sex, greater severity of illness, and longer, more complicated ICU stays. These findings support the need for continued systematic CAM-ICU screening and multidisciplinary delirium-prevention strategies.

## Introduction

Delirium, also known as acute confusional state, is defined by the Diagnostic and Statistical Manual of Mental Disorders, Fifth Edition (DSM-5) as a neuropsychiatric disturbance of acute onset characterized by impaired attention, awareness, and cognition [1]. Its most common manifestations include reduced attentional capacity, short-term memory deficits, disorientation to time, place, and person, sleep-wake cycle disruption, emotional lability, and altered psychomotor behaviour. Symptoms typically fluctuate over the course of the day, often worsening in the evening and at night. Delirium is not better explained by a pre-existing or evolving neurocognitive disorder; rather, it represents the direct physiological consequence of an underlying medical condition, intoxication, substance withdrawal, toxin exposure, or multiple combined aetiologies [1].

The clinical presentation of delirium is heterogeneous and is classically divided into three motoric subtypes: hyperactive, hypoactive, and mixed. Hyperactive delirium is the most readily recognized, characterized by agitation, aggression, or restlessness, and may be accompanied by hallucinations or delusions, which can lead to misdiagnosis as psychosis, hypomania, or an anxiety disorder [2,3]. Hypoactive delirium, the most common subtype in the ICU, is characterized by lethargy, somnolence, and diminished spontaneous movement, and is frequently mistaken for fatigue, dementia, or depression, making it the most difficult subtype to recognize [2,4]. Patients may fluctuate between hyperactive and hypoactive states, a pattern termed mixed delirium [5].

The reported prevalence of delirium varies widely according to the population studied, ranging from roughly 9% to 84% across ICU cohorts [6,7]; a recent systematic review and meta-analysis pooling 33 studies estimated overall ICU delirium prevalence at approximately 33% [8]. Despite this burden, delirium is widely considered underdiagnosed, owing to its fluctuating course, limited awareness among clinical staff, and clinical overlap with dementia [4].

The pathophysiology of delirium remains incompletely understood, but several predisposing and precipitating factors have been consistently associated with its development, including age over 65 years, greater severity of acute illness, psychiatric history, alcohol or substance misuse, and pre-existing sensory impairment. Iatrogenic contributors include prolonged immobilization, sleep-wake cycle disruption, invasive mechanical ventilation, polypharmacy, and use of opioids, benzodiazepines, and anticholinergic agents; nasogastric tubes, urinary catheters, and central venous catheters have also been implicated [3,5,9].

The most widely used severity-of-illness and prognostic scores in the ICU are the Acute Physiology and Chronic Health Evaluation (APACHE), the Simplified Acute Physiology Score (SAPS II) [10], and the Sequential Organ Failure Assessment (SOFA) [9]. The CAM-ICU, a modified version of the original Confusion Assessment Method, is currently the most widely used bedside instrument for delirium detection in the ICU [5]. It is a validated tool that can be administered by trained nursing and medical staff [4,11,12], requires no verbal response from the patient, and can therefore be applied even during invasive mechanical ventilation [13]. In its original validation, the CAM-ICU demonstrated sensitivities of 93% and 100% and specificities of 98% and 100% across two independent raters when compared with a DSM-based reference standard [14]; a subsequent pooled meta-analysis of nine validation studies reported a pooled sensitivity of 80.0% and specificity of 95.9% [12], and the most recent Cochrane review (11 studies, 1,530 patients) reported a pooled sensitivity of 78% and specificity of 95% [13]. The CAM-ICU assesses four features: acute onset or fluctuating course, inattention, disorganized thinking, and altered level of consciousness [2].

Following a diagnosis of delirium, a thorough clinical history and physical examination should be performed to identify reversible contributing causes, most commonly electrolyte disturbances, infection, constipation, urinary retention, dehydration, malnutrition, and medications [3,4]. Delirium during ICU admission has been consistently associated with prolonged length of stay, increased morbidity and mortality, longer duration of mechanical ventilation, higher healthcare costs, and higher hospital readmission rates, as well as complications such as falls, self-extubation, and inadvertent removal of devices [3,5,15,16].

Prevention remains the most effective strategy for reducing the incidence, and consequently the morbidity, mortality, and cost, associated with delirium [4]. Non-pharmacological preventive measures include minimizing nighttime noise and light exposure, avoiding physical restraints, promoting early mobilization, and maintaining adequate hydration [6,16]. First-line management of established delirium is non-pharmacological, comprising reorientation strategies (use of clocks and calendars), emotional support from family and friends, provision of hearing aids or glasses when needed, and sleep hygiene promotion [2,4]. Pharmacological treatment should be reserved for cases refractory to non-pharmacological measures or in which delirium compromises patient safety [4]; most used agents are haloperidol, olanzapine, and quetiapine [2]. Current guidelines favour sedation with propofol or dexmedetomidine over benzodiazepines when sedation is required [17].

Given that delirium is common yet frequently underrecognized in the ICU, we undertook a characterization of this syndrome based on real-world data from a single ICU, in the expectation that this may contribute to a better understanding of the condition, raise clinician awareness, and inform local protocols for delirium prevention and management. The primary objective of this study was to characterize the frequency and clinical profile of CAM-ICU-verified delirium among ICU admissions at a Portuguese community hospital. Secondary objectives were to compare sociodemographic, clinical, and severity-of-illness characteristics between admissions with and without CAM-ICU-verified delirium, and to identify factors independently associated with delirium in a multivariable model.

## Materials and methods

Study design and setting

This was a single-center, retrospective, observational, analytical study conducted in the ICU of the Unidade Local de Saúde do Alto Minho (ULSAM), a Portuguese community hospital.

Study population

All patients admitted to the ICU between January 1, 2021, and December 31, 2022, were eligible. Inclusion criteria were: admission to the ICU during the study period and at least one CAM-ICU assessment recorded in the electronic clinical platform B-ICU.Care® (B-Simple Healthcare Solutions, Porto, Portugal), or a coded diagnosis of delirium in the same platform. Patients younger than 18 years were excluded. A total of 788 ICU admissions, corresponding to 742 individual patients (46 readmissions), met these criteria.

Data collection

Data were extracted from the institutional electronic ICU clinical information system (B-ICU.Care®). They included sociodemographic variables (age, sex), pre-existing comorbidities, admission diagnosis, severity-of-illness scores at admission (APACHE II, SAPS II, SOFA), admission source and type (medical, urgent surgical, elective surgical), CAM-ICU results, coded delirium diagnosis, length of ICU stay, use and duration of invasive mechanical ventilation, and vital status at ICU discharge and at 28 days.

Definitions

Delirium was defined as at least one positive CAM-ICU assessment recorded at any point during the ICU stay. Per institutional practice, CAM-ICU is not attempted in patients with a Richmond Agitation-Sedation Scale (RASS) score of -4 or -5 (deep sedation/unresponsive); assessments are not recorded for these periods, and they were not classified as CAM-ICU-negative. The primary comparative analysis was restricted to admissions with a positive CAM-ICU assessment independently traceable in the electronic nursing records; the rationale for this restriction is described in the Results. The 10 coded-only admissions (a delirium diagnosis coded in the clinical information system without an independently traceable CAM-ICU assessment) were retained in the comparator ("no delirium") group in the primary analysis, since they did not meet the primary CAM-ICU-verified case definition.

Statistical analysis

Categorical variables are described as absolute (n) and relative (%) frequencies. For continuous variables, normality was assessed with the Kolmogorov-Smirnov test; given the known over-sensitivity of this test in large samples, the final classification as normally or non-normally distributed was based on the combined visual inspection of histograms and skewness/kurtosis coefficients rather than the p<0.05 threshold alone [18]. All continuous variables in this study were ultimately classified as non-normally distributed and analyzed using non-parametric methods. Normally distributed variables are reported as mean (M) and standard deviation (SD); non-normally distributed variables are reported as median (Mdn) and interquartile range (IQR).

Patients were divided into two groups (delirium and no delirium). Continuous variables were compared using the independent-samples t-test (normal distribution) or the Mann-Whitney U test (non-normal distribution); effect size was estimated with eta-squared (η²) for the t-test and with r (z/√N) for the Mann-Whitney U test [19]. Categorical variables were compared using the chi-square test, with the Phi coefficient (Φ) reported for 2×2 tables and Cramér's V (φc) for larger tables [20,21]. ICU admissions, rather than unique patients, were used as the unit of analysis throughout; the 46 readmissions (5.8% of admissions) were therefore treated as independent observations.

To estimate the probability of delirium associated with each candidate predictor, we fitted a multivariable binary logistic regression model. Candidate predictors (age, sex, SOFA, and APACHE II) were prespecified a priori based on their established, consistent association with delirium risk in the critical care literature [4,9,22,23] and their availability as routinely collected variables at ICU admission across the cohort: age and sex are fundamental demographic factors implicated in most delirium risk models, while SOFA and APACHE II were selected as the two routinely calculated measures of acute illness severity in this ICU, capturing complementary aspects of physiological derangement and organ dysfunction. Admission type, although modestly associated with delirium in univariable analysis, was not included as an independent covariate because it functions primarily as a proxy for the underlying reason for ICU admission rather than a direct physiological risk factor, and was considered conceptually redundant with the severity-of-illness scores already included. We assessed collinearity among predictors using the variance inflation factor (VIF). Model calibration was assessed using the Hosmer-Lemeshow goodness-of-fit test [21], and discrimination was assessed using the area under the receiver operating characteristic curve (AUC); the model was developed for explanatory rather than predictive purposes and has not been externally validated. A two-tailed p<0.05 was considered statistically significant throughout, with 95% confidence intervals reported where applicable.

Ethical considerations

This study was reviewed and approved by the Ethics Committee for Research in Life and Health Sciences of the University of Minho (CEICVS, approval no. CEICVS 176/2023). All procedures were conducted in accordance with the principles of the Declaration of Helsinki and the Convention on Human Rights and Biomedicine. The authors declare no conflicts of interest.

## Results

General characteristics of ICU admissions

Between January 2021 and December 2022, 788 ICU admissions were identified, corresponding to 742 individual patients (46 readmissions, 5.8%). The sociodemographic and clinical characteristics of the cohort are summarized in Table 1.

**Table 1:** Sociodemographic characterization of the cohort and personal history n: absolute frequency; %: relative frequency; Mdn: median; IQR: interquartile range; COPD: chronic obstructive pulmonary disease.

Variable	Total (%)
Sex (n=742)	
Male	446 (60.1%)
Female	296 (39.9%)
Age, years, Median (IQR) (n=742)	69.0 (21.0)
Personal history (n=742)	
Cardiovascular risk factors	
Hypertension	202 (27.2%)
Diabetes mellitus	105 (14.1%)
Dyslipidaemia	141 (19.0%)
Obesity	78 (10.5%)
Smoking	36 (4.8%)
Alcohol misuse	44 (5.9%)
Comorbidities	
Atrial fibrillation	58 (7.8%)
Chronic liver disease	35 (4.7%)
Chronic kidney disease	34 (4.6%)
COPD	27 (3.6%)
Heart failure	32 (4.2%)
Myocardial infarction	26 (3.5%)
Stroke	21 (2.8%)
Epilepsy	18 (2.2%)
Asthma	12 (1.6%)
Psychiatric history	
Depression	26 (3.5%)
Dementia	6 (0.8%)

The cohort included 446 men (60.1%) and 296 women (39.9%); median age was 69.0 years (IQR 21.0), ranging from 18 to 93 years. Regarding age distribution, 4.3% (n=32) of patients were 18-30 years old, 12.4% (n=92) were 31-50 years old, 36.9% (n=274) were 51-70 years old, 46.0% (n=341) were 71-90 years old, and 0.4% (n=3) were older than 90 years. Regarding cardiovascular risk factors, 27.2% (n=202) had hypertension, 14.1% (n=105) had diabetes mellitus, 19.0% (n=141) had dyslipidaemia, and 10.5% (n=78) had obesity. Regarding lifestyle factors, 4.8% (n=36) were current smokers and 5.9% (n=44) had a history of alcohol misuse. The most frequent comorbidity was atrial fibrillation (7.8%, n=58).

Factors related to ICU admission

Table 2 summarizes admission characteristics. Most admissions originated from the emergency department (49.2%, n=385), followed by the intermediate care unit (20.3%, n=159) and surgical wards (18.8%, n=147); the remainder originated from medical wards (8.8%, n=69), obstetrics and gynaecology (2.6%, n=20), and psychiatry (0.3%, n=2). Regarding type of admission, most episodes were medical (69.5%, n=544), followed by urgent surgery (19.7%, n=154) and elective surgery (10.9%, n=85).

**Table 2:** Characterization of ICU admission source and type n: absolute frequency; %: relative frequency.

Variable	Total
Source of admission (n=782; 6 not recorded)	
Emergency department	385 (49.2%)
Intermediate care unit	159 (20.3%)
Surgical ward	147 (18.8%)
Medical ward	69 (8.8%)
Obstetrics/Gynecology	20 (2.6%)
Psychiatry	2 (0.3%)
Type of admission (n=783; 5 not recorded)	
Medical	544 (69.5%)
Urgent surgery	154 (19.7%)
Elective surgery	85 (10.9%)

Severity scores at ICU admission

Table 3 summarizes severity-of-illness scores. The APACHE II score was incomplete in 68 episodes (8.6%); among the 720 completed scores, the median value was 14.0 (IQR 11.0), with a median predicted mortality of 16.0% (IQR 30.0). The SAPS II score was incomplete in 76 episodes (9.6%); among the 712 completed scores, the median was 34.5 (IQR 23.3), with a median predicted mortality of 18.0% (IQR 28.0). The SOFA score at admission was available in 696 episodes (88.3% of the cohort); the median value was 5.0 (IQR 6.0).

**Table 3:** Severity-of-illness scores at ICU admission (n=788 admissions) Mdn: median; IQR: interquartile range; APACHE: Acute Physiology and Chronic Health Evaluation; SAPS II: Simplified Acute Physiology Score [10]; SOFA: Sequential Organ Failure Assessment [9].

Score	Complete	Incomplete	Value, Median (IQR)	Predicted mortality, %, Median (IQR)
APACHE II	720 (91.4%)	68 (8.6%)	14.0 (11.0)	16.0 (30.0)
SAPS II	712 (90.4%)	76 (9.6%)	34.5 (23.3)	18.0 (28.0)
SOFA at admission	696 (88.3%)	92 (11.7%)	5.0 (6.0)	-

Length of stay, ventilation, and outcomes

ICU length of stay ranged from 1 to 104 days (median 3.0, IQR 5.0); most admissions (606/788, 76.9%) lasted 7 or fewer days, while 58/788 (7.4%) lasted 15 days or more; length of stay was not recorded for five admissions (0.6%). Invasive mechanical ventilation status was recorded for 756 of 788 admissions (32 missing, 4.1%); of these, 42.2%(n=319/756) admissions were ventilated, with a median duration of 3.0 days (IQR 5.0) (Table 4).

**Table 4:** Length of stay, mechanical ventilation, sedative/analgesic use, and vital status n: absolute frequency; %: relative frequency; Mdn: median; IQR: interquartile range.

Variable	Total
ICU length of stay, days (n=788; 5 not recorded)	
Median (IQR)	3.0 (5.0)
1-7 days	606 (76.9%)
8-14 days	119 (15.1%)
≥15 days	58 (7.4%)
Invasive mechanical ventilation (n=756; 32 status not recorded)	
Ventilated	319 (42.2%)
Duration, days, Median (IQR)	3.0 (5.0)
Sedative/analgesic use (n=859 drug-episodes)	
Fentanyl	315 (36.7%)
Propofol	285 (33.2%)
Dexmedetomidine	149 (17.3%)
Midazolam	110 (12.8%)
Vital status at ICU discharge (n=742)	
Improved	503 (67.8%)
Deteriorated	11 (1.5%)
Died	150 (20.2%)
Stable	78 (10.5%)
Vital status at 28 days after ICU admission (n=742)	
Alive	544 (73.3%)
Died	177 (23.9%)
No information	21 (2.8%)

Regarding vital status at ICU discharge (patient level, last admission for readmitted patients), 503 patients (67.8%) were discharged improved, 78 (10.5%) were discharged in stable condition, 11 (1.5%) had deteriorated, and 150 (20.2%) died during ICU admission, corresponding to an ICU mortality rate of 20.2%. 28-day mortality was 23.9% (n=177); this includes those who died during the index ICU stay; vital status at 28 days could not be determined for 21 patients (2.8%).

Regarding sedative and analgesic use during the ICU stay, data was available in 859 drug-episodes. 36.7% (n=315) drug-episodes corresponded to fentanyl, 33.2%(n=285) to propofol, 17.3%(n=149) to dexmedetomidine, and 12.8% n=110) to midazolam; because individual patients could receive more than one agent, these proportions describe drug-episodes rather than the percentage of patients exposed to each drug.

Delirium in the ICU cohort

Over the two-year study period, 5,360 nursing CAM-ICU assessments were recorded, corresponding to approximately 6.8 assessments per admission. Of these, 66 patients (66 admissions) had at least one positive CAM-ICU assessment during their ICU stay, representing 8.4% of admissions (66/788). A further 23 admissions carried a coded diagnosis of delirium in the clinical information system, 13 of which overlapped with the CAM-ICU-positive group, yielding a combined "delirium" definition of 76/788 admissions (9.6%). Because the additional 10 coded-only admissions could not be independently verified against the electronic nursing records, the comparative analysis below is restricted to the 66 admissions with a directly verifiable positive CAM-ICU assessment. Of the 788 admissions, 778 (98.7%) had at least one CAM-ICU assessment independently traceable in the nursing records (66 positive and 712 negative throughout); the remaining 10 (1.3%) entered the cohort solely via a coded delirium diagnosis without a directly traceable CAM-ICU record (see Materials and Methods section for the handling of periods of deep sedation). We prespecify the directly verifiable CAM-ICU-positive definition (8.4%, n=66) as the primary case definition, used consistently in the comparative and regression analyses below; the combined definition (9.6%, n=76) is reported descriptively only.

Comparison between patients with and without delirium

Table 5 compares the characteristics of admissions with and without delirium (CAM-ICU-positive, n=66, vs CAM-ICU-negative, n=722).

**Table 5:** Comparison of admissions with and without delirium (delirium defined as ≥1 positive CAM-ICU) Mdn: median; IQR: interquartile range; Φ: Phi coefficient; φc: Cramér’s V; r: Mann-Whitney effect size (z/√N); APACHE: Acute Physiology and Chronic Health Evaluation; SAPS II: Simplified Acute Physiology Score; SOFA: Sequential Organ Failure Assessment.

Variable	Delirium (n=66)	No delirium (n=722)	Effect size	p
Sex, female	14 (21.2%)	298 (41.3%)	Φ = 0.114	p = 0.001
Age, years, Median (IQR)	71.0 (18.0)	68.0 (21.0)	r = 0.08	p = 0.021
Admission type			φc = 0.105	p = 0.013
- Medical	39 (59.1%)	505 (69.9%)		
- Urgent surgery	22 (33.3%)	132 (18.3%)		
- Elective surgery	5 (7.6%)	80 (11.1%)		
APACHE II, Median (IQR) (completed)	18.5 (10.5) (n=66)	14.0 (12.0) (n=654)	r = 0.11	p = 0.002
SAPS II, Median (IQR) (completed)	43.0 (18.8) (n=66)	34.0 (24.0) (n=646)	r = 0.12	p = 0.001
SOFA at admission, Median (IQR)	7.0 (5.5) (n=63)	5.0 (7.0) (n=633)	r = 0.10	p = 0.010
Mechanical ventilation	40/64 (62.5%)	279/692 (40.3%)	Φ = 0.125	p = 0.001
- Duration, days, Median (IQR)	4.5 (6.5) (n=40)	3.0 (4.0) (n=279)	r = 0.12	p = 0.032
ICU length of stay, days, Median (IQR)	8.0 (8.0)	3.0 (4.0)	r = 0.24	p < 0.001
Status at ICU discharge			φc = 0.107	p = 0.028
- Improved	53 (80.3%)	490 (67.9%)		
- Stable	7 (10.6%)	77 (10.7%)		
- Deteriorated	2 (3.0%)	9 (1.2%)		
- Died	4 (6.1%)	146 (20.2%)		

Patients who developed delirium were more frequently male (78.8%; n=52/66) than patients without delirium (58.7%; n=424/722); this association was statistically significant (χ²=10.18, Φ=0.114, p=0.001). Regarding age, patients with delirium had a higher median age (71.0 years, IQR 18.0) than those without delirium (68.0 years, IQR 21.0; Mann-Whitney p=0.021, r=0.08). Admission type differed modestly between groups (χ²=8.68, Cramér's V=0.105, p=0.013), with a higher proportion of urgent surgical admissions among patients with delirium.

Severity-of-illness scores at admission were all higher in patients who developed delirium. The median completed APACHE II score was 18.5 (IQR 10.5) in the delirium group versus 14.0 (IQR 12.0) in the non-delirium group (p=0.002, r=0.11). The median SAPS II score was 43.0 (IQR 18.8) versus 34.0 (IQR 24.0; p=0.001, r=0.12). The median SOFA score at admission was 7.0 (IQR 5.5) versus 5.0 (IQR 7.0; p=0.010, r=0.10).

Patients with delirium were more frequently mechanically ventilated (62.5%; n=40/64) than those without delirium ( 40.3%; n= 279/692; χ²=11.82, Φ=0.125, p=0.001); among ventilated patients, those with delirium also had a longer median duration of ventilation (4.5 vs 3.0 days, p=0.032, r=0.12). The temporal relationship between mechanical ventilation and delirium detection could not be established in this dataset; these findings should be interpreted as associations rather than as evidence that ventilation is a risk factor for, or a consequence of, delirium.

ICU length of stay was substantially longer in patients with delirium (median 8.0 days, IQR 8.0) than in those without (median 3.0 days, IQR 4.0; Mann-Whitney p<0.001, r=0.24), the largest effect size observed among the variables studied. This association is likely bidirectional: longer ICU stays increase the opportunity for CAM-ICU assessment and for delirium to develop, while delirium itself is a recognized contributor to prolonged critical illness; causal directionality cannot be inferred from this cross-sectional comparison.

Status at ICU discharge differed modestly between groups (χ²=9.11, Cramér's V=0.107, p=0.028); ICU mortality was numerically lower among patients with delirium (6.1%; n=4/66) than among those without (20.2%; n=146/722). This pattern is consistent with survivorship/immortal-time bias: among admissions that died in the ICU, 24.7% of those without delirium died within 24 hours of admission and 48.6% within 72 hours, compared with none of the four deaths in the delirium group (median time to death 14.4 days), indicating that many early deaths occurred before there was a realistic opportunity to accumulate a positive CAM-ICU assessment. A sensitivity analysis restricting the comparison to admissions that survived at least three days, thereby excluding admissions with limited opportunity for CAM-ICU assessment, showed a materially similar, still statistically significant difference (delirium: 7.3% n=4/55 vs no delirium: 20.8% n= 75/360; χ²=5.68, p=0.017), indicating that survivorship bias, while clearly present in the timing pattern, does not fully account for the observed mortality difference. This finding should nonetheless be interpreted cautiously given the small number of deaths in the delirium group.

Predictors of delirium

Table 6 presents the results of the multivariable logistic regression model (n=696 admissions with complete data for all four predictors; 63 delirium events).

**Table 6:** Multivariable logistic regression: predictors of delirium (n=696; 63 delirium events; Hosmer-Lemeshow χ²=7.67, p=0.466) CI: confidence interval; SOFA: Sequential Organ Failure Assessment [9]; APACHE: Acute Physiology and Chronic Health Evaluation.

Predictor	Odds ratio (OR)	95% CI	p
Age (per year)	1.03	1.00-1.05	p = 0.017
Male sex (ref. female)	3.09	1.58-6.03	p = 0.001
SOFA at admission (per point)	1.02	0.95-1.10	p = 0.505
APACHE II (per point)	1.02	0.99-1.05	p = 0.214

Older age was independently associated with delirium (OR 1.03 per additional year, 95% CI 1.00-1.05, p=0.017). Male sex was independently associated with a higher probability of delirium relative to female sex (OR 3.09, 95% CI 1.58-6.03, p=0.001; equivalently, female sex was associated with an OR of 0.32 relative to male sex). Neither the SOFA score (OR 1.02, p=0.505) nor the APACHE II score (OR 1.02, p=0.214) reached statistical significance in the adjusted model. The Hosmer-Lemeshow goodness-of-fit test did not indicate lack of fit (χ²=7.67, p=0.466), supporting adequate model calibration. Collinearity was not a concern: VIF values ranged from 1.01 (male sex) to 1.58 (APACHE II), with age at 1.10 and SOFA at 1.50, all well below thresholds indicative of problematic multicollinearity. Model discrimination was modest (AUC=0.68), consistent with this model's explanatory, rather than predictive, purpose. A sensitivity analysis restricted to each patient's first ICU admission only (n=742 patients, 61 delirium events; n=656 with complete predictor data) yielded materially similar conclusions: age (OR 1.02, 95% CI 1.00-1.05, p=0.030) and male sex (OR 2.77, 95% CI 1.43-5.38, p=0.003) remained independently associated with delirium, while SOFA (OR 1.02, p=0.628) and APACHE II (OR 1.02, p=0.200) remained non-significant, indicating that the inclusion of readmissions as independent observations did not materially alter our conclusions.

## Discussion

Principal findings

This study identified 66 ICU admissions with at least one positive CAM-ICU assessment among 788 admissions over a two-year period, a frequency of 8.4%, toward the lower end of the range reported in the literature, including a large international multicenter study reporting a pooled incidence near 32% [6,8,24]; case definitions and ascertainment methods vary substantially across studies, which limits direct comparability. This relatively low figure should be interpreted cautiously. The institutional protocol recommends CAM-ICU assessment at least once per nursing shift (three times daily), which would imply roughly 12 assessments over a median 4-day stay; the observed average of 6.8 assessments per admission is lower than this. Part of this gap reflects appropriate, protocol-directed non-assessment during periods of deep sedation (Richmond Agitation-Sedation Scale ≤-4), when CAM-ICU is not attempted; however, we cannot fully exclude some degree of incomplete screening adherence beyond this, which would tend to under-ascertain delirium. This is at least as plausible an explanation for the low frequency observed as any genuine difference in delirium occurrence. A large multicenter study similarly found that "routine", nurse-driven CAM-ICU screening detects substantially fewer cases than research-grade assessment, illustrating how screening intensity and consistency can influence measured frequency independent of the true underlying occurrence [25]. Regular, structured training on delirium recognition and management is provided to nursing and medical staff at our institution by a dedicated "Sedation, Analgesia and Delirium" Working Group, and it is possible that this and systematic screening contribute to genuinely earlier recognition and management of delirium, as has been proposed elsewhere [3]; however, this retrospective, single-center study, without a comparator period or site, cannot distinguish between these explanations, and we present the training/prevention hypothesis as speculative rather than established.

Several non-pharmacological delirium-prevention strategies are also already embedded in this ICU's routine practice, aimed at preserving sleep quality and the sleep-wake cycle: exposure to natural light through windows installed in patient bays [26], reduced nighttime artificial lighting ("lights-off" hours and individual bedside lighting to minimize disruption when artificial light is required), noise-level monitoring, and nighttime earplugs for patients who tolerate them [27]. Flexible, extended visiting hours and the presence of personal items (glasses, watches, books, photographs) are also permitted. Similar bundled non-pharmacological strategies allowed Malik et al. to report a 20% relative reduction in delirium incidence in a randomized trial [28].

Predictors of delirium

In this cohort, male sex and older age were independently associated with delirium. The association with age is well established and consistent with the broader literature [4,9]. The association with male sex is less uniformly reported: some cohorts have found male sex associated with a higher incidence of delirium, while others report the opposite or no association, and a recent narrative review characterized the evidence on sex-specific differences in ICU delirium as limited and somewhat contradictory [29]. A large Medical Information Mart for Intensive Care IV (MIMIC-IV)-based analysis of patients who had already developed ICU delirium found that women subsequently had higher short-term mortality than men, despite lower rates of invasive ventilation and vasoactive support before delirium onset, raising the possibility of sex-related disparities in the recognition or treatment of delirium that merit further study [29]. Our finding of higher unadjusted and adjusted odds of delirium in men should be interpreted as hypothesis-generating; it was not anticipated a priori, and larger, ideally multicenter, cohorts with granular exposure data (sedation strategy, physical restraint use, family visitation) would be needed to explore potential mediators.

Regarding severity-of-illness scores at admission, patients who developed delirium had significantly higher APACHE II, SAPS II, and SOFA scores than those who did not, consistent with the broader literature linking greater acute physiological derangement to delirium risk. Lobo-Valbuena et al. [22], in a prospective observational study, found that higher SAPS-3 and SOFA scores were associated with delirium; Rahimi-Bashar et al. [23] similarly reported an association between higher APACHE scores and delirium risk in a prospective cohort. A prior systematic review by Krewulak et al., however, highlighted inconsistent findings across studies regarding the association between severity scores and delirium risk, and called for greater standardization in the collection and reporting of these scores [30]. Our findings align with the more consistent recent evidence that greater illness severity at admission is associated with delirium [22,30].

Patients with delirium in our cohort also had a markedly longer ICU stay (median 8 vs 3 days) and were more frequently and more prolongedly mechanically ventilated than those without delirium, findings that are highly consistent with the broader literature associating delirium with worse ICU-resource utilization and are also biologically and clinically plausible: delirium both predisposes to, and results from, prolonged critical illness and its treatments (sedation, immobilization, sleep disruption) [6,16]. Admission type and vital status at ICU discharge also differed modestly between groups (both p<0.05, with small effect sizes, Cramér's V ≈0.11). Prior work has highlighted substantial heterogeneity across studies in the reported association between these admission-level variables and delirium risk [30]. Data on alcohol misuse could not be examined as a covariate in this analysis, as this variable was not available in sufficient detail in the electronic records (see Limitations).

Current guidelines favor sedation with propofol or dexmedetomidine over benzodiazepines [17]. In this cohort, fentanyl and propofol were the two most frequently recorded agents, with more limited use of benzodiazepines such as midazolam; our data do not permit confirmation of concurrent co-administration at the patient level. Given the well-described association between benzodiazepine exposure and delirium in the literature [5], it is possible that the relatively limited recorded use of benzodiazepines in this cohort contributed to the observed frequency; however, this remains speculative, as detailed dose, timing, and pre-delirium exposure data were not available for systematic analysis. Although not the primary focus of this study, and in the absence of a systematic record of therapeutic choices, routine management of delirium in this ICU follows current recommendations, combining individualized non-pharmacological measures with pharmacological agents (dexmedetomidine, olanzapine, haloperidol, and quetiapine) when required.

Limitations

This study is observational and retrospective and therefore relies on data recorded for clinical rather than research purposes, with the attendant limitations of incomplete records, heterogeneity among the clinicians documenting care, and unmeasured confounding. Because the exact timing of CAM-ICU assessments relative to ICU admission was not extracted, we could not distinguish delirium present at or shortly after admission (prevalent) from delirium developing later during the stay (incident); the frequency reported here reflects the overall proportion of admissions with at least one positive assessment during the stay. Screening adherence could also not be fully verified beyond the RASS-related non-assessment described in the Methods, and incomplete screening may have contributed to under-ascertainment of delirium. Because admissions rather than patients were used as the unit of analysis, the 46 readmissions (5.8%) were not statistically independent of one another, which could modestly underestimate standard errors; a sensitivity analysis restricted to one (first) admission per patient yielded materially similar results (see Results). Retaining the 10 coded-only admissions in the "no delirium" group may have introduced non-differential misclassification if some represented true, undetected delirium.

Several variables relevant to delirium risk were not captured, including pre-existing visual or hearing impairment and use of glasses or hearing aids during ICU stay. Sleep quality during ICU admission would also have been a relevant variable, but no validated protocol for its assessment exists in this ICU. Detailed data on sedative and analgesic dosing were similarly not available for systematic analysis. This single-center study was conducted in a peripheral community hospital, which may explain the comparatively small number of delirium admissions and may limit generalizability. The modest number of CAM-ICU-positive admissions also limits statistical power for some comparisons and the precision of effect estimates, particularly for less common outcomes such as ICU mortality.

## Conclusions

The frequency of delirium identified in this cohort (8.4% by CAM-ICU criteria) was lower than most previously reported figures. Older age and, notably, male sex were independently associated with the development of delirium during ICU admission, alongside greater severity of illness at admission and a substantially longer, more complicated ICU course. Delirium management is multidisciplinary and combines non-pharmacological and pharmacological measures. This ICU has already implemented several non-pharmacological strategies targeting sleep-wake cycle preservation and sleep quality, together with systematic, at least once-per-shift CAM-ICU screening of every patient. The implementation of a structured screening tool such as the CAM-ICU, together with multidisciplinary delirium-prevention and management strategies, is likely to help reduce the burden of this common and consequential ICU complication. Given the novelty of the sex-delirium association observed in this cohort, external replication in a larger, multicenter cohort is warranted.

## References

[1] American Psychiatric Association (2013). Diagnostic and Statistical Manual of Mental Disorders, Fifth Edition.

[2] Stollings JL, Kotfis K, Chanques G, Pun BT, Pandharipande PP, Ely EW (2021). Delirium in critical illness: clinical manifestations, outcomes, and management. Intensive Care Med.

[3] Zanobini F, Nitti M, Giubbolini G, Giubbolini F, Fagiolini A (2017). Early recognition and treatment of delirium using the Confusion Assessment Method for the Intensive Care Unit (CAM-ICU) in a cardiac surgical intensive care unit. Eur Neuropsychopharm.

[4] Marcantonio ER (2017). Delirium in hospitalized older adults. N Engl J Med.

[5] Poulsen LM, Estrup S, Mortensen CB, Andersen-Ranberg NC (2021). Delirium in intensive care. Curr Anesthesiol Rep.

[6] Salluh JI, Wang H, Schneider EB (2015). Outcome of delirium in critically ill patients: systematic review and meta-analysis. BMJ.

[7] Girard TD, Jackson JC, Pandharipande PP (2010). Delirium as a predictor of long-term cognitive impairment in survivors of critical illness. Crit Care Med.

[8] Wu NN, Zhang YB, Wang SY, Zhao YH, Zhong XM (2023). Incidence, prevalence and risk factors of delirium in ICU patients: a systematic review and meta-analysis. Nurs Crit Care.

[9] Jeong IS, Cho MK (2023). Factors affecting delirium in ICU patients. Int J Environ Res Public Health.

[10] Le Gall JR, Lemeshow S, Saulnier F (1993). A new Simplified Acute Physiology Score (SAPS II) based on a European/North American multicenter study. JAMA.

[11] Ely EW, Margolin R, Francis J (2001). Evaluation of delirium in critically ill patients: validation of the Confusion Assessment Method for the Intensive Care Unit (CAM-ICU). Crit Care Med.

[12] Gusmao-Flores D, Salluh JI, Chalhub RÁ, Quarantini LC (2012). The confusion assessment method for the intensive care unit (CAM-ICU) and intensive care delirium screening checklist (ICDSC) for the diagnosis of delirium: a systematic review and meta-analysis of clinical studies. Crit Care.

[13] Miranda F, Gonzalez F, Plana MN, Zamora J, Quinn TJ, Seron P (2023). Confusion Assessment Method for the Intensive Care Unit (CAM-ICU) for the diagnosis of delirium in adults in critical care settings. Cochrane Database Syst Rev.

[14] Ely EW, Inouye SK, Bernard GR (2001). Delirium in mechanically ventilated patients: validity and reliability of the confusion assessment method for the intensive care unit (CAM-ICU). JAMA.

[15] Oh ES, Fong TG, Hshieh TT, Inouye SK (2017). Delirium in older persons: advances in diagnosis and treatment. JAMA.

[16] Ely EW, Shintani A, Truman B (2004). Delirium as a predictor of mortality in mechanically ventilated patients in the intensive care unit. JAMA.

[17] Devlin JW, Skrobik Y, Gélinas C (2018). Clinical practice guidelines for the prevention and management of pain, agitation/sedation, delirium, immobility, and sleep disruption in adult patients in the ICU. Crit Care Med.

[18] Kim HY (2013). Statistical notes for clinical researchers: assessing normal distribution (2) using skewness and kurtosis. Restor Dent Endod.

[19] Field AP (2017). Discovering Statistics Using IBM SPSS Statistics, 5th edition.

[20] McHugh ML (2013). The chi-square test of independence. Biochem Med (Zagreb).

[21] Hosmer DW, Lemeshow S, Sturdivant RX (2013). Applied Logistic Regression.

[22] Lobo-Valbuena B, Gordo F, Abella A (2021). Risk factors associated with the development of delirium in general ICU patients. A prospective observational study. PLoS One.

[23] Rahimi-Bashar F, Abolhasani G, Manouchehrian N, Jiryaee N, Vahedian-Azimi A, Sahebkar A (2021). Incidence and risk factors of delirium in the intensive care unit: a prospective cohort. Biomed Res Int.

[24] Salluh JI, Soares M, Teles JM (2010). Delirium epidemiology in critical care (DECCA): an international study. Crit Care.

[25] van Eijk MM, van den Boogaard M, van Marum RJ (2011). Routine use of the confusion assessment method for the intensive care unit: a multicenter study. Am J Respir Crit Care Med.

[26] Lee HJ, Bae E, Lee HY, Lee SM, Lee J (2021). Association of natural light exposure and delirium according to the presence or absence of windows in the intensive care unit. Acute Crit Care.

[27] Van Rompaey B, Elseviers MM, Van Drom W, Fromont V, Jorens PG (2012). The effect of earplugs during the night on the onset of delirium and sleep perception: a randomized controlled trial in intensive care patients. Crit Care.

[28] Malik AK, Baidya DK, Anand RK, Subramaniam R (2021). A new ICU delirium prevention bundle to reduce the incidence of delirium: a randomized parallel group trial. Indian J Crit Care Med.

[29] Schreiber N, Eichlseder M, Orlob S (2024). Sex specific differences in short-term mortality after ICU-delirium. Crit Care.

[30] Krewulak KD, Stelfox HT, Ely EW, Fiest KM (2020). Risk factors and outcomes among delirium subtypes in adult ICUs: a systematic review. J Crit Care.

